# Molecular mechanisms related to bone damage in spinal tuberculosis revealed by 4D-label-free proteomics analysis

**DOI:** 10.3389/fcimb.2025.1629805

**Published:** 2025-09-22

**Authors:** Wenxuan Xiao, Guangling Yang, Shuqin Xu, Shu Song, Yuting Tang, Tianyao Zhou, Weili Huang, Lu Zhang, Yutong Gu

**Affiliations:** ^1^ Department of Microbiology, School of Life Science, Fudan University, Shanghai, China; ^2^ Department of Orthopaedic Surgery, Zhongshan Hospital, Fudan University, Shanghai, China; ^3^ Shanghai Public Health Clinical Center, Fudan University, Shanghai, China; ^4^ Shanghai Southwest Spine Surgery Center, Shanghai, China

**Keywords:** spinal tuberculosis, *Escherichia coli* infection, 4D-label free proteomics, bone metabolism, molecular mechanism, bone damage

## Abstract

**Aims:**

Spinal tuberculosis (STB) is a common form of extrapulmonary tuberculosis (ETB). However, the molecular mechanism of pathological injury in STB remains unclear. The purpose of this study was to explore the pathogenic mechanism of STB, and compare it with *Escherichia coli* (E.coli) bone infections and lumbar degenerative disease (LDD) patients.

**Main methods:**

In this study, the infected lumbar spine bone tissue of STB patients was collected for the infection group. LDD patients and E.coli lumbar spine infection (SEcoli) patients were collected for the non-M.TB infection group. Proteins from the bone tissue were extracted for 4D-Label Free Proteomics (4D-LFQ) analysis to compare the pathogenesis and immune mechanisms of STB and SEcoli.

**Key findings:**

The osteoclast growth inhibitory factors tumor necrosis factor receptor superfamily member 11B (TNFRSF11B) and semaphorin-3A (Sema3A) were significantly down-regulated in STB, while the protein Wnt-5a (WNT5A) secreted by osteoblasts was significantly up-regulated. These changes in STB bone metabolism may lead to an increase in the number of osteoclasts and bone injury. In addition, the significantly up-regulated expression of thymocyte selection-related family member 2 (THEMIS2) suggests that THEMIS2 may be a potential therapeutic target for STB that could control the Toll-like receptor response of macrophages. Meanwhile, the PI3K-Atk anti-apoptotic pathway and the ECM-receptor interaction pathway were inhibited during both infections.

**Significance:**

This study explored the pathogenic mechanism of STB based on proteomics and compared its differences with E.coli bone infection, providing new insight into the treatment of STB.

## 1 Introduction

Tuberculosis (TB) is an infectious disease caused by the bacterium *Mycobacterium tuberculosis* (M.tb). Even in the modern era, with such advanced medical technology, TB remains one of the leading causes of death from a single source of infection and the thirteenth leading cause of death globally. M.tb can cause widespread infections in human tissues and organs, including the lungs, bones, cervical lymph nodes, meninges, peritoneum, intestines, skin, etc. The lung is the most common target site of M.tb, i.e., pulmonary tuberculosis. Extrapulmonary tuberculosis (ETB) accounts for 15% of TB cases, causing significant damage to the body (Navarro-Flores et al., 2022). As one of the most common ETB lesion sites (Gorse et al., 1983; Chung et al., 2014; Jain et al., 2020), spinal tuberculosis (STB) accounts for approximately 1% to 3% of all tuberculosis cases and nearly 50% of musculoskeletal infection cases (Wang et al., 2021). Due to the increasing drug resistance rate in M.tb and the incidence rate of HIV-TB co-infection, the proportion of STB cases is also on the rise (Jin et al., 2014; Chen et al., 2016; Ali et al., 2019). STB usually occurs in adolescents, but children and the elderly may also get infected (Tufan et al., 2005; Kiymaz et al., 2006; Claro-Almea et al., 2020; Jain et al., 2020). Pediatric and elderly spinal tuberculosis requires particularly early attention. The growth characteristics of the spine and the presence of cartilage tissue are the main reasons for certain behavioral differences in spinal tuberculosis between children and adults. The cartilaginous nature of the vertebral bodies in children makes them more susceptible to relatively rapid vertebral destruction (Dimeglio and Canavese, 2012; Chatterjee and Banta, 2018). This explains why deformities in children tend to be more severe and develop over a much shorter period of time compared to adults. Studies have shown that before receiving anti-tuberculosis treatment, the percentage of abscess formation in the elderly is significantly lower than that in the young and middle-aged populations. This may be attributed to stronger immune responses and increased immune cell infiltration in younger patients (Li et al., 2024). Compared to younger patients, elderly patients exhibit significantly higher positive expression rates of TGF-β and elevated levels of IL-10. The combined production of IL-10 and TGF-β may reduce host immunity against Mycobacterium tuberculosis, leading to uncontrolled bacterial replication and progression to overt disease (Bonecini-Almeida et al., 2004; Li et al., 2024).

However, current research on M.tb infection mainly focuses on pulmonary tuberculosis, with few studies on STB. Only a limited number of reports have paid close attention to STB diagnosis (Chen et al., 2016; Tang et al., 2017; Shen et al., 2019; Karthek et al., 2021) and therapeutic approaches (Shi et al., 2012; Jin et al., 2014; Cao et al., 2018). Even fewer studies have explored the molecular mechanisms underlying the occurrence and progression of STB, which are primarily related to macrophages (Huang et al., 2019; Wang et al., 2020; Xu et al., 2021). An insufficient understanding of STB poses obstacles to its prevention, diagnosis and treatment. Therefore, it is necessary to enhance our understanding of the pathogenic and host immune mechanisms specific to STB.

There are many pathogenic bacteria that can cause spinal infections, such as *Staphylococcus aureus* and *Escherichia coli* (E.coli), among others (Matsuura et al., 2019; Muthukrishnan et al., 2019; Masters et al., 2021). To comprehensively understand the molecular changes in STB vertebral lesions and distinguish the unique pathological mechanisms of lumbar spine infection of M.tb and E.coli, this study was the first to extract and compare bone tissue proteomes from STB patients, E.coli lumbar spine infection (SEcoli) patients and lumbar degenerative disease (LDD) patients using 4D-FQ technology. In recent years, advances in mass spectrometry (MS) have established 4D label-free technology as a cutting-edge approach for proteomic analysis (Zhao et al., 2022). Due to its high sensitivity and bioinformatic advantages, this technique enables the identification of potential functional proteins, including many low-abundance proteins that are difficult to detect using conventional methods (Wang et al., 2023; Yang et al., 2023; Ren et al., 2024). However, no previous studies have applied this technology to investigate potential pathogenic mechanisms in bone tissues from clinical patients with spinal tuberculosis or E.coli bone infections.

This research characterized the patterns of bone metabolism, cellular matrix-related biological processes and immune pathways in STB and SEcoli. In STB patients, the bone metabolism changes and inflammatory responses caused osteoclast proliferation, cell apoptosis and reduction of extracellular matrix (ECM), which ultimately led to infected bone damage. We demonstrated the aggregation of macrophages in granuloma tissue and how pathogenic bacteria escape the immune system by inhibiting the apoptosis pathway. Further comparison of the proteome in lumbar spine tissue post M.tb and E.coli infection revealed the molecular pathological specificity of STB.

## 2 Materials and methods

### 2.1 Subjects and ethics statement

For the proteomic analysis of STB vertebrae, samples were obtained from 3 patients with STB and 3 patients with lumbar degenerative disease (LDD). For the proteomic analysis of SEcoli vertebrae, samples were collected from 2 patients with SEcoli and 5 patients with LDD. Bone tissue from LDD patients was used as the control group without infection, rather than as “healthy controls,” due to the unavailability of completely healthy human bone tissue. All samples were clinical bone tissue specimens that were processed as paraffin-embedded sections. These were collected through intraoperative sampling, and these tissue sections were subsequently used for proteomic analysis. Detailed patient information is provided in **Supplementary Table S1**.

To further validate the accuracy and reproducibility of the proteomic data, an additional 10 fresh clinical samples obtained intraoperatively from STB and LDD patients, respectively, were collected for qRT-PCR and immunohistochemical analyses.

The inclusion criteria for STB patients were as follows: 1) Presence of typical symptoms of tuberculous toxemia, such as lassitude, afternoon pyrexia, and night sweats, accompanied by chest, thoracic, or lumbar back pain; 2) Laboratory findings, including positive T-SPOT test results, and elevated ESR and CRP levels; 3) Imaging evidence of corresponding spinal lesions; and 4)intraoperative pathological examination and culture confirming M. tuberculosis infection. Drug susceptibility testing was not performed.

The inclusion criteria for SEcoli patients were as follows: 1) High fever accompanied by chest, thoracic, or lumbar back pain; 2)Laboratory findings, including elevated white blood cell count, ESR, CRP, and a positive blood culture for E. coli; 3) Imaging evidence of corresponding spinal lesions; and 4) Intraoperative pathological examination and culture confirming E. coli infection.

The inclusion criteria for LDD patients were as follows: 1) diagnosis of LDD through MRI; 2) no intervertebral space or bone destruction; and 3) no STB and no SEcoli.

Exclusion criteria: Any of the following conditions were not included in this study: 1) Patients with other diseases that could hurt the spine; 2) Patients having diseases that affect immune function, autoimmune diseases; and 3) patients who have received spinal surgery.

All patients gave written consent for the use of their specimens in this study, which was approved by the Medical Ethics Committee of Zhongshan Hospital, Fudan University (B2021-530R).

### 2.2 Paraffin-embedded tissue samples

The tissues obtained from patients were separated from each other and cleaned with PBS. The different tissues were fixed separately in formalin (Sinopharm Chemical Reagent, China) for 24–48 hours at room temperature. They were then decalcified with 10% EDTA (Sinopharm Chemical Reagent, China), dehydrated with a series of ethyl alcohols (Sinopharm Chemical Reagent, China), cleared with xylene (Sinopharm Chemical Reagent, China) and embedded in paraffin (Sinopharm Chemical Reagent, China) (Mueller et al., 2017; Lee et al., 2021). The paraffin-embedded bone tissue samples were used for 4D-LFQ analysis and Ziehl-Neelsen staining. Granulation paraffin-embedded samples were used for immunohistochemical analysis.

### 2.3 Ziehl-Neelsen staining

The paraffin-embedded bone tissue samples were sectioned, deparaffinized, and stained as previously described (Wang et al., 2020). Briefly, the sections were stained with carbol fuchsin (BASO, China) for 1h at 60 °C, decolorized with acid alcohol (BASO, China) for 30 s, and restained with methylene blue (BASO, China) for 1min at room temperature. After each staining and destaining step, any remaining dyes or reagents on the sections were rinsed with deionized water. Then red acid-fast bacilli were looked for under the optical microscope.

### 2.4 Protein extraction and trypsin digestion

After deparaffinization of paraffin-embedded bone tissue, 4 volumes of lysis buffer (1% SDS, 1% protease inhibitors, Merck Millipore, Germany) were added and then lysed by sonication. Cell debris was removed by centrifugation at 14000g for 15min, after which the supernatant was transferred for protein concentration determination. If the collected protein was enough for 4D-LFQ detection and no protein degradation was found through SDS-PAGE, then we would further perform trypsin digestion. An equal amount of protein (100 μg) was taken from each sample and adjusted to the same volume with lysis buffer. One volume of acetone (pre-cooled at -20 °C) was added and fully mixed and then 4 more volumes of pre-cooled acetone were added. After standing at -20 °C for 2h, the precipitate was collected by centrifugation at 4500g for 5min. The pellet was washed with pre-cooled acetone twice, left to dry, and then dissolved in 200 mM Tetraethylammonium bromide (TEAB, Sigma, US). Trypsin (Promega, China) was added at a ratio of protease: protein of 1:50, and the samples were digested overnight at room temperature. Afterwards, dithiothreitol (DTT, Sigma, US) was added to each sample at a final concentration of 5 mM and the samples were reduced at 56 °C for 30min. Finally, iodoacetamide (IAA) was added to reach a final concentration of 11 mM, and the mixture obtained was incubated at room temperature in the dark for 15 min. 

### 2.5 Liquid chromatography-mass spectrometry (MS)/MS analysis

An EASY-nLC 1200 ultra-high performance liquid chromatography (UHPLC) system was used to separate the peptides. Mobile phase A [0.1% formic acid (Fluka, US) and 2% acetonitrile (ThermoFisher Scientific, US)] and mobile phase B (0.1% formic acid and 90% acetonitrile) for liquid chromatography were prepared first. Peptides were separated with following gradient: 0-14 min, 6%-24%B; 14-16 min, 24%-35%B; 16-18 min, 35%-80%B; 18-20 min, 80%B, and all at a constant flow rate of 500 nl/minon a NanoElute UHPLC system (Bruker Daltonics). After separation by the UHPLC system, the peptides were injected into the Nano-spray ionization (NSI) ion source and analyzed by Orbitrap Exploris™ 480 mass spectrometry. The ion source voltage was set to 2.3 kV and the FAIMS compensation voltage (CV) to -45V, -65V. The high-resolution Orbitrap was also used for the detection and analysis of peptide parent ions and their secondary fragments.

The primary mass spectrometer’s scanning range was set to 400–1200 m/z, and the secondary mass spectrometer’s scanning range was set to a fixed starting point of 110 m/z. The scanning resolutions of the two mass spectrometers were set to 6000 and 15000, respectively. The TurboTMT was turned off on the secondary mass spectrometer. A data-dependent scanning (DDA) program was used to collect data. After the first level of scanning, the peptide precursor ions entered the higher-energy collision dissociation (HCD) cell sequentially, using 27% fragmentation energy, followed by secondary MS. At this point, the system parameter settings for the mass spectrometer were adjusted as follows to improve the effective utilization of MS: automatic gain control (AGC) was set to 100%, signal threshold was set to 5E4 ions/s, maximum injection time was set to auto, and dynamic exclusion time for tandem mass spectrometry scans was set to 20 s.

### 2.6 Protein functional enrichment

Gene Ontology (GO) enrichment analysis was performed. GO annotations of proteins are divided into three categories: biological processes (BP), cellular components (CC) and molecular functions (MF). Protein annotation analysis was performed with the EggNOG-mapper software (v2.0) based on the EggNOG database. Fisher’s exact test was used to analyze the significance of the GO enrichment in differentially expressed proteins, where a P value < 0.05 was considered significant.

Kyoto Encyclopedia of Genes and Genomes (KEGG) pathway enrichment analysis was performed. Protein pathways were annotated based on the KEGG pathway database. The identified differentially expressed proteins were subjected to BLAST alignment (blastp, e-value ≤ 1e-4), and the annotations were selected based on the highest alignment scores. Fisher’s exact test was also used here to analyze the significance of the differentially expressed proteins pathways, where a P value < 0.05 was considered significant.

### 2.7 Quantitative real-time PCR

The clinical bone tissues were rinsed immediately with saline and stored at −80 °C for RNA extraction. With the aid of sterile surgical forceps, the bone tissues were crushed into small pieces or powder, and then ground in a mortar with liquid nitrogen. For every 10 mg of bone tissue, 1 mL of TRIzol reagent (Invitrogen, USA) was added. After thorough grinding, the mixture was transferred to grinding tubes for RNA extraction. Subsequently, 200 μL of chloroform was added to each tube, which was vigorously shaken for 30 s to fully mix the contents and facilitate RNA separation. The samples were then centrifuged at 12,000 rpm for 20 min at 4 °C. The upper aqueous phase was carefully transferred to 1.5 mL RNase-free EP tubes. An equal volume of pre-chilled isopropanol was then added to each tube, and the mixture was shaken vigorously for 30 s, followed by centrifugation at 12,000 rpm for 20 min at 4 °C. The supernatant was discarded, and the RNA pellet was washed twice with pre-cooled (−30 °C) 75% ethanol. After removing the ethanol, the RNA samples were obtained. According to the manufacturer’s instructions, 1 μg of RNA was reverse transcribed into cDNA using a cDNA synthesis kit (Takara, Japan). The qRT-PCR reaction system consisted of 0.1 μL of cDNA, 0.7 μL each of forward and reverse primers (10 μM), 5 μL of 2× Talent qRT Premix, 0.2 μL of 50× Rox Dye, and RNase-free H_2_O to a final volume of 10 μL. GAPDH was used as the reference gene. The calculation method for the mRNA expression levels of each gene was demonstrated by Liang Wang et al (Wang et al., 2020). The primer sequences are shown in **Supplementary Table S2**, and the qRT-PCR program is shown in **Supplementary Table S3**.

### 2.8 Immunohistochemical and immunofluorescence verification

Deparaffinized paraffin sections of granuloma tissue samples from patients with STB were immersed in a citrate buffer solution (pH 6.0), followed by heating in a microwave oven for antigen retrieval. After blocking the endogenous peroxidase with a 3% hydrogen peroxide solution, the sections were blocked with a 3% BSA (Solarbio, China). The sections were incubated overnight at 4 °C with a primary antibody against CD68 (1:200, CST, Germany). After washing with PBS (pH 7.4), a secondary antibody (DAKO, K5007 kit, Denmark) was added, and the sections were incubated for 50 min. The DAB chromogenic solution (1:100, DAKO, K5007 kit, Denmark), stained the positive area brown-yellow. The sections were then stained with hematoxylin, differentiated in 1% hydrochloric acid alcohol and blued with ammonia. In this way, nuclear regions of the sections were stained blue. For immunofluorescence staining, the samples were incubated overnight at 37°C with a primary antibody against CD68, TGF-β, IL-6, and caspase-3 (1:200, CST, Germany). After washing with PBS (pH 7.4), a secondary antibody (1:1000, Abcam, UK) was added and the sections were incubated for 60min. Fluorescent imaging was performed using a confocal microscope. The samples were imaged with an Olympus confocal microscope FV3000. iMaris x64 9.0.1 software was used to perform fluorescence and quantitative image analyses.

### 2.9 Statistical analysis

Statistical analysis was performed using GraphPad Prism 8. The difference between the two groups was analyzed using a t-test analysis. Bar graphs with error bars represent the mean ± standard deviation (SD). Significance is indicated by asterisks: * P ≤ 0.05, **P ≤ 0.01, ***P ≤ 0.001, ****P ≤ 0.0001.

## 3 Results

### 3.1 Comparative proteomic analysis of lumbar spine tissues in STB and LDD patients

**Figure 1:**
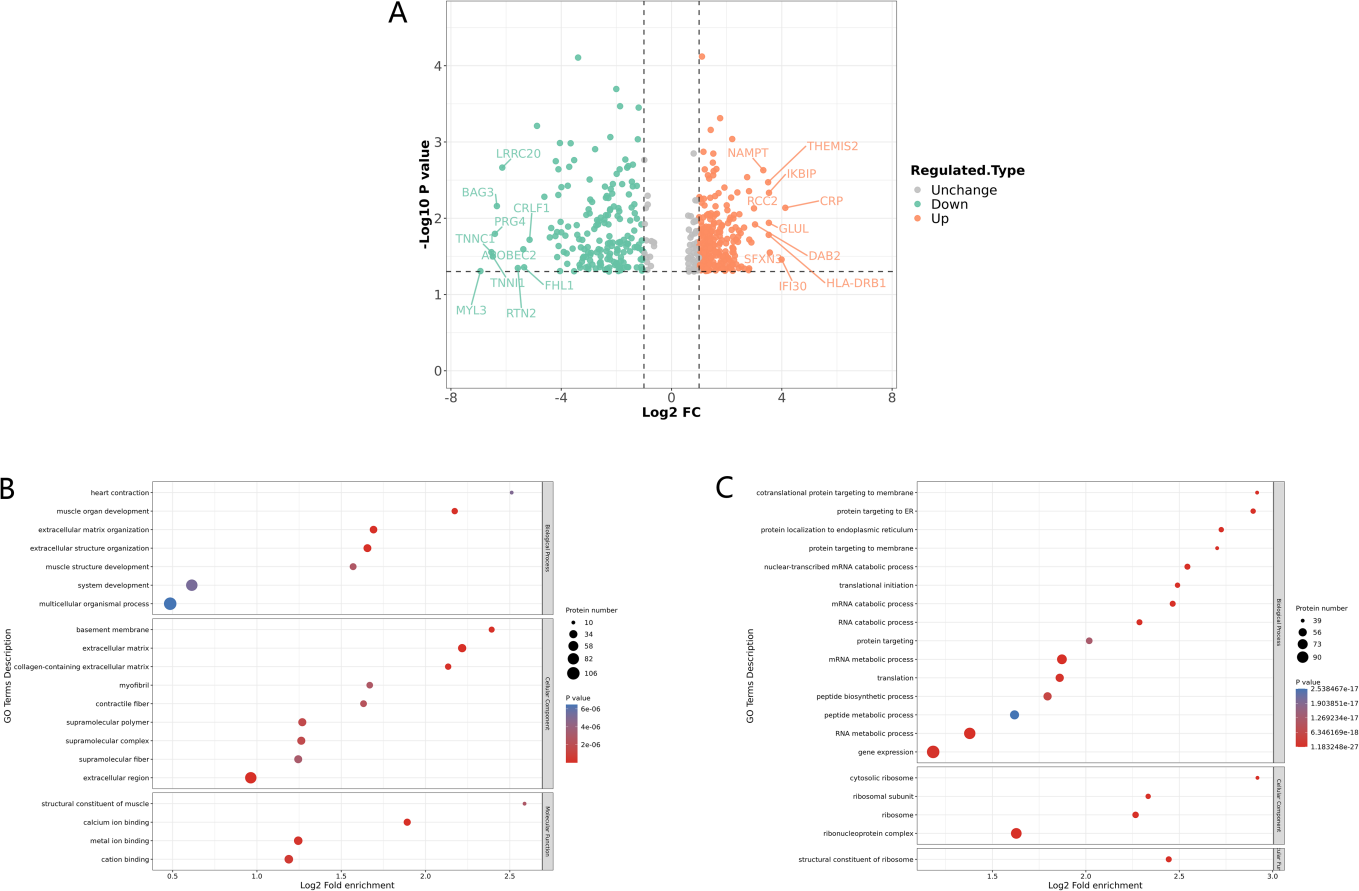
Identification and GO enrichment analysis of differentially expressed proteins in STB. **(A)** Differential protein volcano plot: proteins with a p value < 0.05 and a fold change > 1.2 are considered differentially expressed proteins. Red dots represent up-regulated differentially expressed proteins, green dots represent down-regulated differentially expressed proteins, and gray dots represent proteins with no differential expression. The red marked proteins are the ten most up-regulated proteins with the greatest difference in p value, while the green marked proteins are the ten most down-regulated proteins with the greatest difference in p value; **(B, C)** GO enrichment analysis of down-regulated **(B)** and up regulated **(C)** differentially expressed proteins, the greater the number of enriched proteins, the larger the circle. The size of p-value is represented by color and presented on the left side of the graph. From red to blue of the circle, p-value increases gradually.

The lumbar and thoracic vertebrae, being relatively weight-bearing and mobile parts of the spine, are the two areas with the highest incidence of STB (Cao et al., 2018). Lumbar STB patients with definite symptoms of M.tb infection were selected for this research whose MRI images showed obvious damage to the lumbar vertebrae and disc (**Supplementary Figure S1A**; **Supplementary Table S1**). The existence of bacilli with a size of approximately 3 μm was revealed by Ziehl-Neelsen staining in the infected bone tissues obtained by surgery (**Supplementary Figure S1B**). 4D-FLQ proteomics analysis of lumbar spine tissues was then conducted on 3 STB and 3 LDD samples, and a total of 3252 quantifiable proteins were identified. Of these, 481 were differentially expressed proteins (P-value <0.05), including 283 that were significantly up-regulated and 198 that were significantly down-regulated. The cutoff values for the fold change and P value were set to 2 and 0.05 respectively. In STB patients, the three up-regulated proteins with the greatest difference multiple were CRP (ratio=17.4641, P-value = 0.0073), IFI30 (ratio=15.9002, P-value = 0.0348) and SFXN3 (ratio=11.8254 ratio, P-value = 0.0282), while the three down-regulated proteins with the greatest difference multiple were MYL3 (ratio=0.0082, P-value = 0.0493), TNNC1 (ratio=0.0108, P-value = 0.0279) and TNNI1 ( ratio=0.0112, P-value = 0.0317) (**Figure 1A**).

Meanwhile, GO enrichment analysis of the differentially expressed proteins showed that the muscle development processes were significantly down-regulated, including muscle organ development, muscle system processes, muscle structure development, and structural constituents of muscle, among others (**Figure 1B**). In addition, it is worth noting that extracellular matrix organization (ECM) was also significantly down-regulated (**Figure 1B**), as ECM plays an important role in maintaining the stability of bone tissue structure, and a reduction in ECM may lead to the destruction of vertebrae and intervertebral disc tissue (Kaplan and Levenberg, 2022). We also found that proteins related to gene expression, mRNA and RNA metabolic processes, and translation were significantly up-regulated (**Figure 1C**).

### 3.2 Macrophage aggregation, phagocytosis and cell apoptosis were promoted at the site of lumbar injury in STB patients

**Figure 2:**
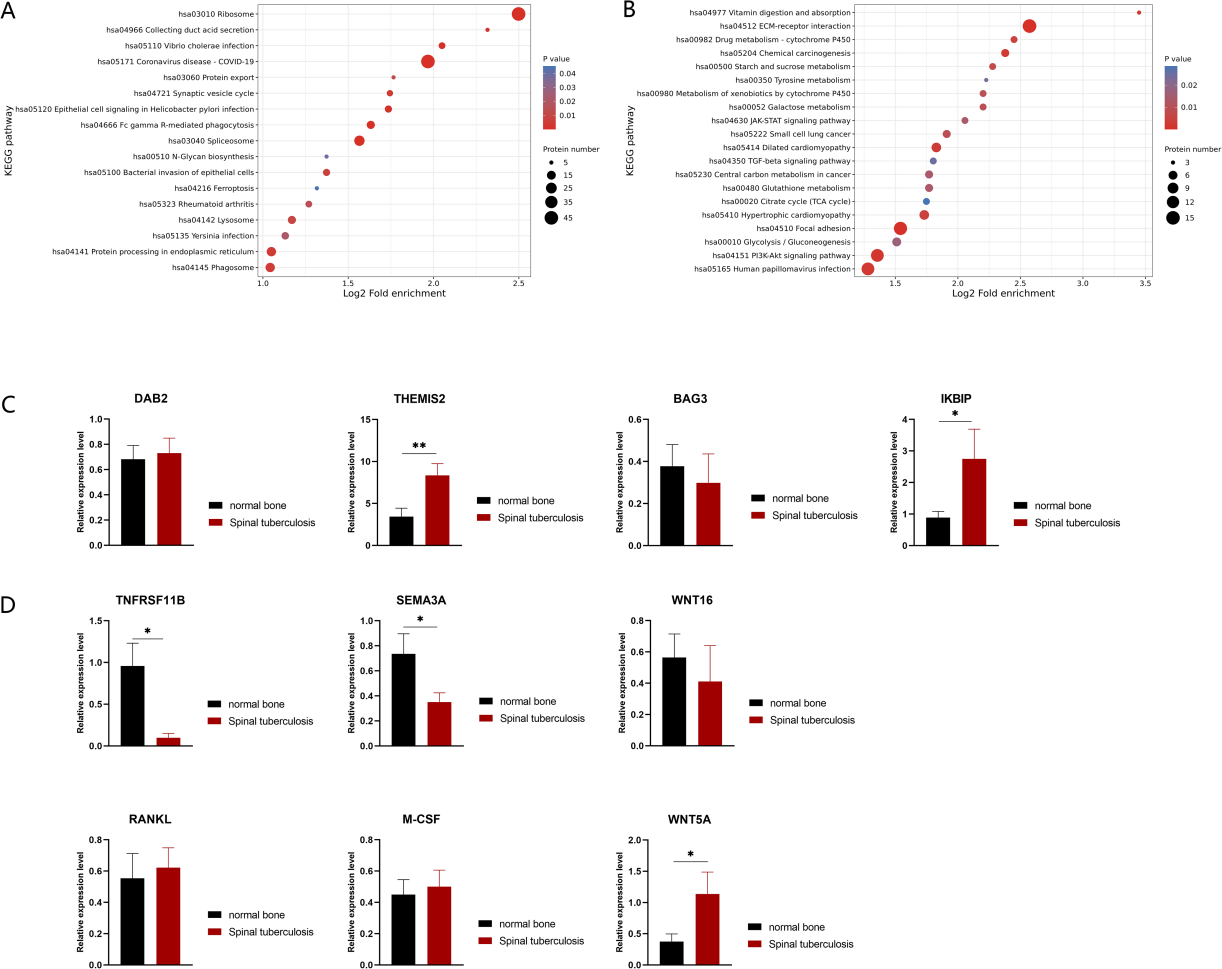
KEGG enrichment of differentially expressed proteins in STB and validation by qRT-PCR. **(A)** Up-regulated differentially expressed proteins enriched KEGG pathways; **(B)** down-regulated differentially expressed proteins enriched KEGG pathways. The greater the number of enriched proteins, the larger the circle. From red to blue of the circle, p value increases gradually. **(C, D)** Validation of differences in mRNA expression changes of proteins by qRT-PCR, n=10. Significant differences between groups determined by T-test are shown: *P < 0.05; **P < 0.01.

M.tb infection can trigger antiviral or immune responses in host cells (Cohen et al., 2018). To identify the specific host defense responses of STB patients to M.tb infection, we focused on the immune response processes associated with these differentially expressed proteins. KEGG enrichment analysis revealed that the up-regulated expression proteins were mainly enriched in the Fc gamma R-mediated phagocytosis and phagosome pathways (**Figure 2A**). Correspondingly, the macrophage marker CD68 was detected by staining for macrophage infiltration in the granuloma tissue of STB patients, which demonstrated the aggregation of large numbers of macrophages at infection sites (**Supplementary Figure S2**). The proteins DAB2 (ratio=8.1590, P-value = 0.0121) and THEMIS2 (ratio=11.3424, P-value = 0.0034), were significantly up-regulated with high fold change and both were found to be related to macrophage function. Protein THEMIS2 is mainly expressed in macrophages and B cells and can act as a signaling scaffold for macrophages, selectively regulating TNF expression downstream of TLR4 (Peirce et al., 2010). qRT-PCR showed that the mRNA expression level of protein THEMIS2 (P-value=0.0085) (**Figure 2C**) was also significantly increased.

Some down-regulated proteins were found enriched in the JAK-STAT and PI3K-Akt signaling pathways (**Figure 2B**), and they were found to exert anti-apoptotic effects through translation regulation (Xin et al., 2020) and inhibit apoptosis by phosphorylating target proteins through various downstream pathways (Sun et al., 2020). The down-regulation of these pathways suggests that massive cell apoptosis may occur in diseased bone tissue. Notably, BAG3 (ratio=0.0124, P-value = 0.0069) an anti-apoptotic protein, was significantly down-regulated with the fifth largest fold change. IKBIP (ratio=11.5836, P-value = 0.0046), one of the up-regulated proteins with the largest fold change, was also found to have a pro-apoptotic function (Hofer-Warbinek et al., 2004). Although there was no statistically significant difference in BAG3 transcriptional levels (P-value=0.6450) (**Figure 2C**), the mRNA expression levels of IKBIP (P-value=0.0481) (**Figure 2C**) were significantly increased in STB samples. This further supports the existence of extensive cell apoptosis in infected bones.

### 3.3 Bone metabolism and the balance between osteoblasts and osteoclasts were damaged in STB patients

The balance between the metabolism and development of osteoblasts and osteoclasts determines the stability and function of bone tissue. Through comparative proteomic analysis, we observed a significant decrease in osteoclast inhibitory factors, TNFRSF11B (also known as OPG, ratio=0.1646, P-value=0.0437) and Sema3A (ratio=0.0469, P-value=0.0178) in STB samples. In this case, the expression levels of 13 bone metabolism-related genes were further tested by qRT-PCR, including the coding genes of *TNFRSF11B* and *Sema3A* (**Figure 2D**). It has been reported that osteoblasts can induce osteoclasts through factors such as receptor activator of nuclear factor kappa-B ligand (RANKL), macrophage colony-stimulating factor 1 (M-CSF) and protein Wnt-5a (WNT5A). The mRNA levels of these osteoclastogenic factors showed upward trends in STB samples (**Figure 2D**). Conversely, osteoblasts can also inhibit osteoclast activity through factors such as TNFRSF11B, Sema3A and Protein Wnt-16 (WNT16) (Han et al., 2018), which showed downward trends in STB samples at the mRNA level. Of these, TNFRSF11B and Sema3A were down-regulated, and WNT5A was up-regulated with statistical significance. The expression patterns of these proteins suggest that the number of osteoclasts in the infected bone of STB may be higher than that of non-infected bone tissue and disrupting the balance of bone metabolism could cause damage to the spine.

### 3.4 Comparative proteomic analysis of lumbar spine tissue in SEcoli and LDD patients

**Figure 3:**
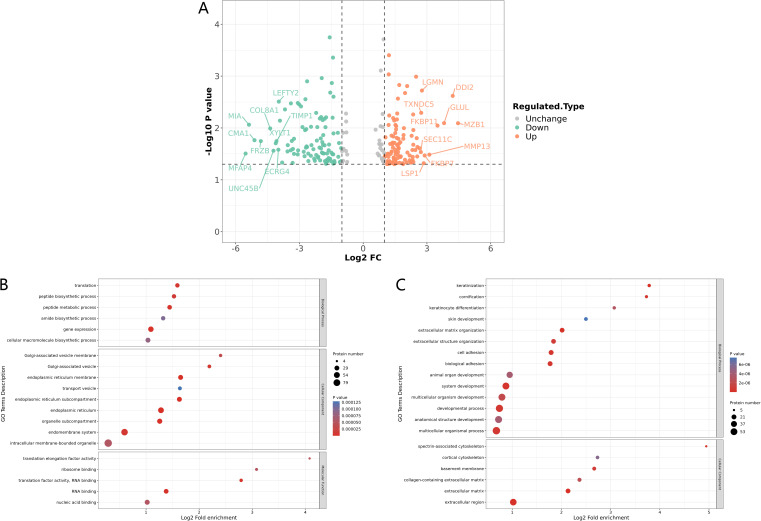
Identification and GO enrichment analysis of differentially expressed proteins in SEcoli. **(A)** Differential protein volcano plot: proteins with a p value < 0.05 and a fold change > 1.2 are considered differentially expressed proteins. Red dots represent up-regulated differentially expressed proteins, green dots represent down-regulated differentially expressed proteins, and gray dots represent proteins with no differential expression. The red-marked proteins are the ten most up-regulated proteins with the greatest difference in p value, while the green marked proteins are the ten most down-regulated proteins with the greatest difference in p value; **(B, C)** GO enrichment analysis of up-regulated **(B)** and down-regulated **(C)** differentially expressed proteins. The greater the number of enriched proteins, the larger the circle. From red to blue of the circle, p-value increases gradually. .

Clinically, in addition to Mtb, there are many pathogenic microorganisms that can cause lumbar spine infections, including E.coli. Regrettably, few studies have been reported on SEcoli, and the mechanism has not yet been clarified to date. We chose 2 SEcoli patients whose lumbar spine had been infected with E.coli and 4 LDD patients as a non-infectious lumbar spine control group. The differences in their bone tissue proteomic characteristics were depicted through 4D-LFQ, and a total of 3346 quantifiable proteins were identified. A total of 237 differentially expressed proteins were detected, of which 124 were significantly up-regulated and 113 were significantly down-regulated. The cutoff values for fold change and P value were set to 2 and 0.05 respectively. Among the SEcoli samples, the three up-regulated proteins with the most significant differences were found to be MZB1 (ratio=22.0385, P-value = 0.0081), DDI2 (ratio=18.5559, P-value = 0.0024) and GLUL (ratio=13.9853, P-value = 0.0081), while the three down-regulated proteins with the largest change in differential expression fold were CMA1 (ratio=0.0289, P-value = 0.0172), FRZB (ratio=0.0355, P-value = 0.0179) and MIA (ratio=0.0241, P-value = 0.0087) (**Figure 3A**). 

Furthermore, GO enrichment analysis showed that the processes of gene expression, translation, transport and RNA binding were up-regulated (**Figure 3B**). The processes related to muscle injury responses, including musculature, muscle growth and other muscle developmental processes, were significantly down-regulated. In addition, the response to bone morphogenetic protein (BMP), regulation of wound response, and TGF-β binding functions were also significantly downregulated in SEcoli samples (**Figure 3C**).

**Figure 4:**
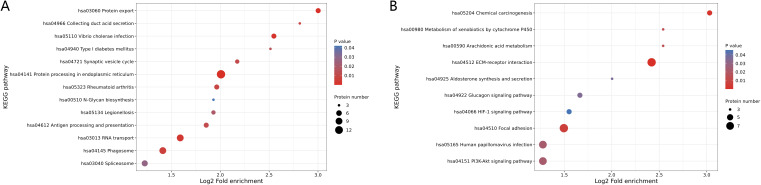
KEGG enrichment of differentially expressed proteins in SEcoli. **(A)** Up-regulated differentially expressed proteins enriched KEGG pathways; **(B)** down-regulated differentially expressed proteins enriched KEGG pathways. The greater the number of enriched proteins, the larger the circle. From red to blue of the circle, p-value increases gradually.

The KEGG enrichment analysis showed that the up-regulated proteins in the SEcoli samples were significantly enriched in the pathways of phagosome, protein processing in the endoplasmic reticulum, RNA transport and protein export pathways (**Figure 4A**), while the down-regulated proteins were enriched in ECM-related pathways (**Figure 4B**), including MFAP4 (ratio=0.0216, P-value=0.0311), VWF (ratio=0.2310, P-value=0.0359), LAMA5 (ratio=0.3360, P-value=0.0002), VTN (ratio=0.5020, P-value=0.028), and TNC (ratio=0.2760, P-value=0.031). It is worth mentioning that the down-regulated proteins in SEcoli were significantly enriched in anti-apoptotic pathways, such as the PI3K-akt pathway (**Figure 4B**), and there was significant up-regulation of pro-apoptotic factors, such as IKBIP (ratio=5.4873, P-value=0.0267). This implies that E.coli infection leads to apoptotic phenomena similar to those of M.tb infection. 

### 3.5 Comparing differentially expressed proteins expressed in the lumbar spine related to M.tb and E.coli infection

**Figure 5:**
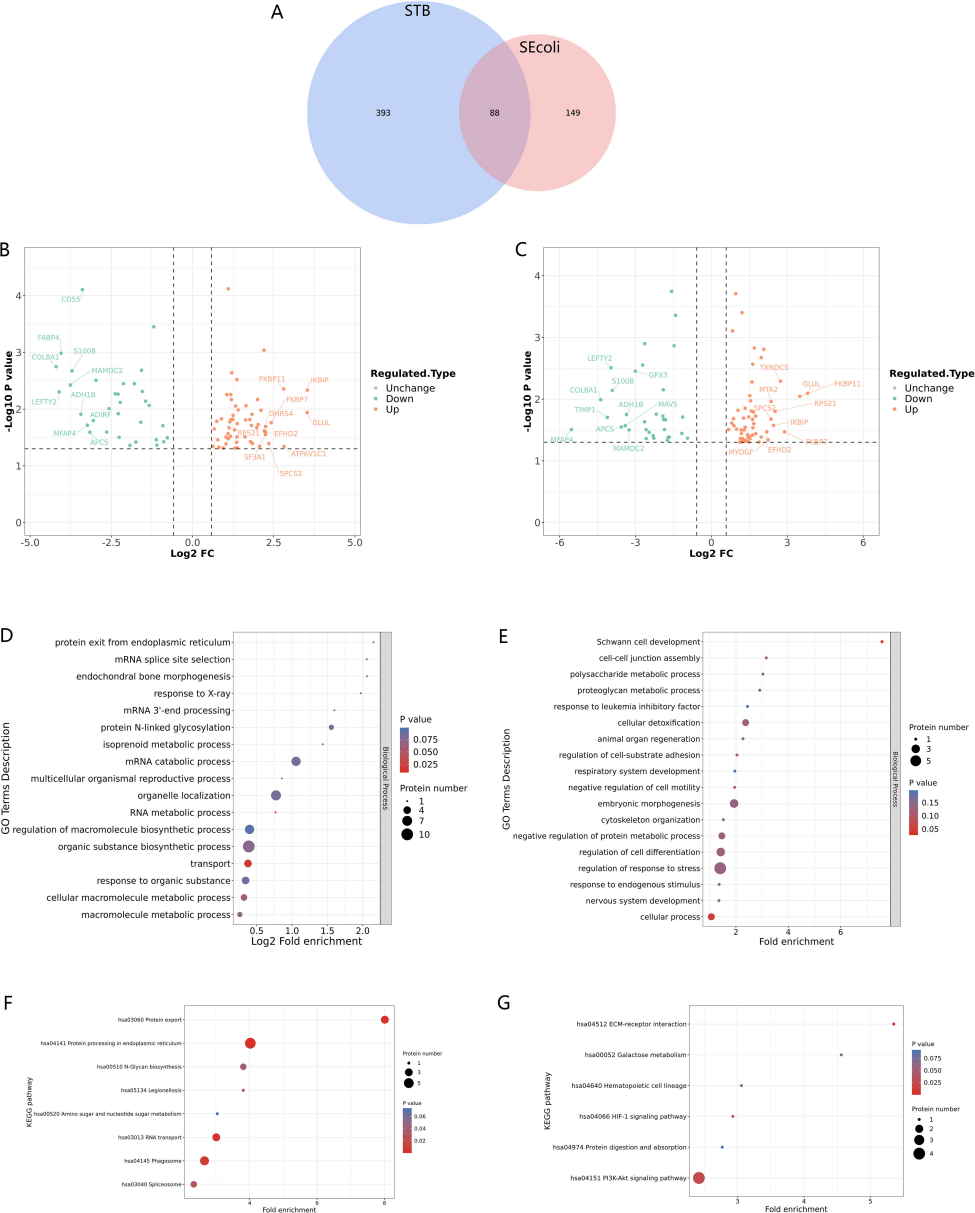
Identification and GO/KEGG enrichment analysis of differentially expressed proteins in STB and SEcoli. **(A)** Wayne diagram showing the overlap of differentially expressed proteins identified in STB and SEcoli; **(B, C)** Volcano plots showing the differentially expressed proteins in STB (left) and SEcoli (right). Proteins with a p value < 0.05 and a fold change > 1.2 are considered differentially expressed. Red dots represent up-regulated differentially expressed proteins, green dots represent down-regulated differentially expressed proteins, and gray dots represent proteins with no differential expression. Red marked proteins are the ten most up-regulated proteins with the greatest difference in p-value, while the green marked proteins are the ten most down-regulated proteins with the greatest difference in p-value; **(D, E)** The GO enrichment analysis represents the up-regulated **(D)** and down-regulated **(E)** differentially expressed proteins respectively. The greater the number of enriched proteins, the larger the circle. From red to blue of the circle, p value increases gradually; **(F, G)** The KEGG enrichment analysis represents the up-regulated **(F)** and down-regulated **(G)** differentially expressed proteins, respectively. The greater the number of enriched proteins, the larger the circle. From red to blue of the circle, p-value increases gradually.

To gain insight into the molecular mechanisms of lumbar spine injuries specific to different pathogenic bacterial infections, we compared differentially expressed proteins identified in STB and SEcoli samples. First, we sorted out and analyzed the differentially expressed proteins with the same trends in both STB and SEcoli samples to reveal common changes in the lumbar spine after infection by pathogenic bacteria. There were 88 identical differentially expressed proteins in the two infection samples (**Figure 5A**). The volcano plot showed the 10 proteins with the greatest fold changes in the STB and SEcoli samples, with overlap in the up-regulated proteins FKBP7, SPCS2, and GLUL, and in the down-regulated proteins S100B, ADH1B, MFAP4, and COL8A1 (**Figures 5B, C**).

GO analysis showed that these common differentially expressed proteins were enriched in biological processes, rather than cellular composition or molecular function (**Figures 5D, E**). Up-regulated proteins were enriched in the process of RNA metabolism and transcription processes, while down-regulated proteins were mainly concentrated in cellular processes. Specifically, the up-regulated differentially expressed proteins were mainly concentrated in endoplasmic reticulum protein processing, phagosomes, and RNA transport, while the down-regulated proteins were mainly concentrated in the PI3K-Atk anti-apoptosis pathway and the ECM-receptor interaction pathway (**Figures 5F, G**), suggesting that M.tb or E.coli infection might disrupt the ECM and inhibit apoptosis to escape host immune clearance.

**Figure 6:**
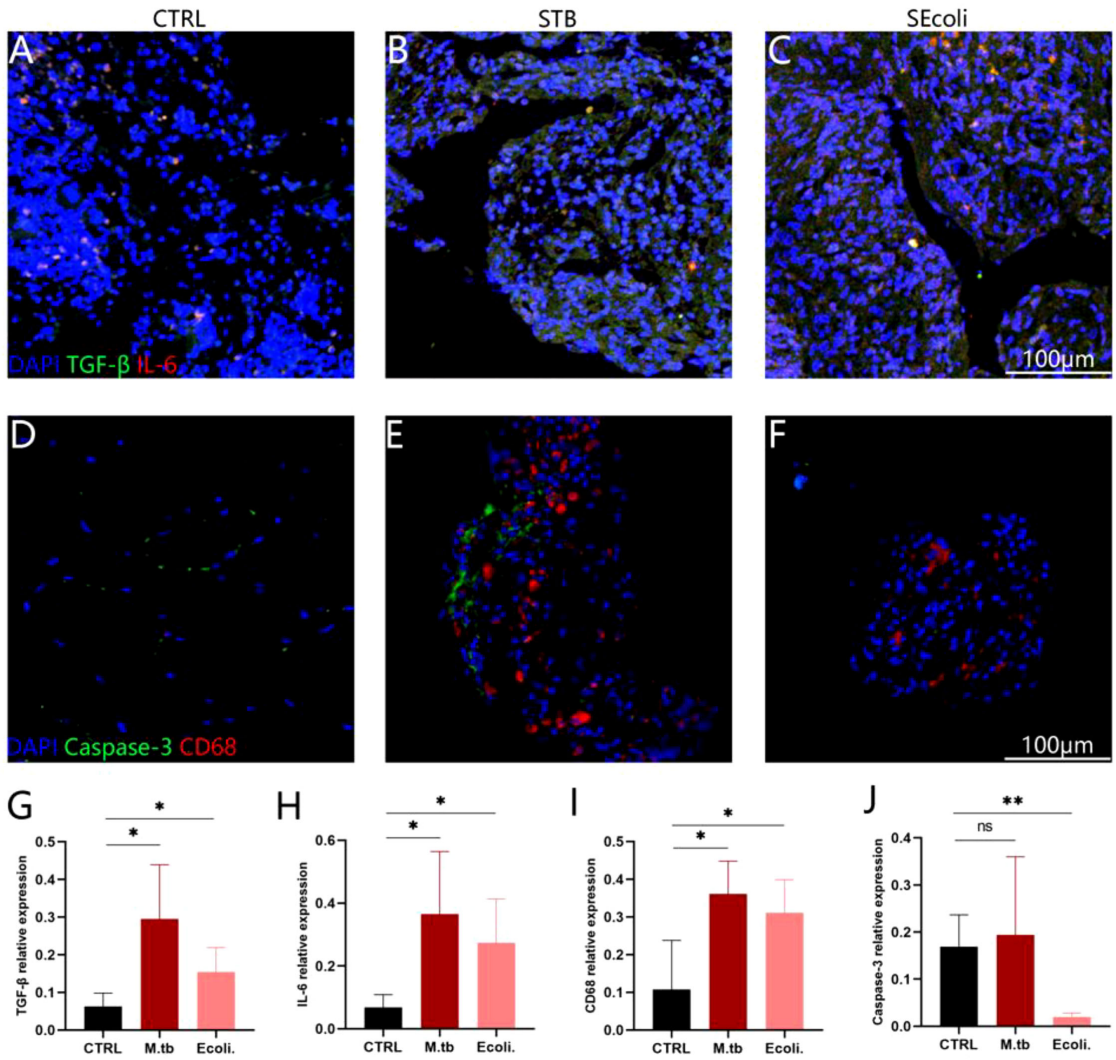
Immunofluorescence staining of human bone tissue. **(A-C)** Staining the nuclei (DAPI, blue), TGF-β (green), IL-6 (red) in healthy subjects, in patients with a lumbar tuberculosis infection and an E.coli lumbar infection, respectively, n=4; **(D-F)** Staining the nuclei (DAPI, blue), caspase-3 (green), and CD68 (red) in healthy subjects, in patients with a lumbar tuberculosis infection and an E.coli lumbar infection, respectively, n=4; **(G-J)** Quantification of the relative fluorescence intensity. The relative fluorescence intensity represents the total fluorescence intensity of the target protein/the total fluorescence intensity of the DAPI, n=4. Significant differences between groups determined by T-test are shown: *P < 0.05; **P < 0.01.

BMP is a member of the TGF-β superfamily that affects the formation of bone and cartilage. TGF-β and BMP signaling disorders are usually associated with osteoarthritis in human and mouse models (Wu et al., 2024). We further validated the inflammatory response caused by bone injury through immunofluorescence. The results showed that TGF-β expression was elevated in both STB and SEcoli (**Figures 6A-J**). Moreover,CD68 and IL-6 staining showed macrophage aggregation and an inflammatory response in both STB and SEcoli (**Figures 6A-J**).

Next, we analyzed the differentially expressed proteins specific to STB and SEcoli samples to reveal the specific molecular mechanisms underlying pathologic injury to the lumbar spine caused by Mtb infection. Interestingly, the up-regulated proteins (MZB1, DDI2) and the down-regulated proteins (CMA1, FRZB, MIA) with the maximum fold change in SEcoli samples were not found in STB samples (**Figure 3A**). Similarly, the three proteins (CRP, IFI30, and SFXN3) with the maximum upregulation and the three proteins (MYL3, TNNC1, and TNNI1) with the maximum down-regulation in STB were not found in SEcoli samples (**Figure 1A**). Notably, the differentially expressed proteins related to osteoblasts and osteoclasts identified in the STB samples (TNFRSF11B, Sema3A, WNT16, RANKL, M-CSF, and WNT5A) were not present in SEcoli, suggesting that these may represent unique molecular mechanisms underlying the bone damage caused by M.tb infection. 

Studies have shown that high-level expression of GSDME leads to the occurrence of pyroptosis (Liao et al., 2022), and we indeed found that the expression of GSDME (ratio=5.0104, P-value=0.0416) was up-regulated in SEcoli cases, but not in STB cases. However, immunofluorescence showed that the expression of Caspase-3 in SEcoli was significantly decreased, while the expression of Caspase-3 in STB was not significantly changed (**Figures 6A-C, G**).

## 4 Discussion

zAs one of the most common ETB manifestations, normally STB coexists with or is secondary to pulmonary tuberculosis (Colmenero et al., 2013); however, the number of cases of primary STB is increasing. Insufficient treatment can lead to serious spinal instability and deformity, possibly involving the nervous system. So it is necessary to optimize the STB diagnosis and treatment methods. Enhancing the understanding of the molecular mechanisms of STB is a prerequisite for achieving this goal. There are already several mechanistic studies on STB (Chernyaeva et al., 2018; Wang et al., 2020; Xu et al., 2021), but the bone metabolism or immune characteristics related to the bone damage after M.tb infection are far from clear.

In this study, we found that the bone damage caused by bacterial infection may be the combined result of decreased ECM expression, down-regulation of TNFRSF11B and Sema3A expression, up-regulation of WNT5A expression, and increased cell apoptosis. ECM not only participates in maintaining bone tissue homeostasis, but is also a necessary substrate for axonal attachment and growth (Kaplan and Levenberg, 2022). The reduction of ECM in diseased vertebrae may partly explain the neural damage primarily caused by mechanical compression at the molecular level. In addition, as a chronic wasting disease, the majority of patients with STB suffer from loss of appetite and reduced diet, which may cause a stunted condition (Putra et al., 2021). The significant down-regulation of muscle development-related proteins reflects the molecular mechanisms behind these symptoms. Without appropriate medical intervention and nutrient intake, more serious damage to the body’s motor function may occur.

Studies have verified that macrophages massively infiltrate the surrounding tissues of caseous necrosis, and that the signaling pathways related to antigen recognition and macrophage phagocytosis are significantly enriched in the early stages of STB (Wang et al., 2020). We also found significant macrophage aggregation in granuloma tissue, and notable enrichment of up-regulated differentially expressed proteins in the Fc gamma R-mediated phagocytosis and phagosome KEGG signaling pathways in bone tissue. All these findings confirm the irreplaceable role of macrophages in defending against M.tb infection of the spine. In addition, we observed that the protein THEMIS2 was significantly up-regulated in diseased bone tissue. As a novel macrophage signaling scaffold, protein THEMIS2 acts as a possible control point for inflammatory responses in macrophages, and is a potential target for intervening in chronic inflammatory diseases involving TNF, such as rheumatoid arthritis (Peirce et al., 2010). Our study suggests the possibility of THEMIS2 as a therapeutic target for spinal tuberculosis. 

There are many pathogenic bacteria that infect lumbar bone tissue, such as *Staphylococcus aureus* and E.coli, leading to serious bone damage, including spinal epidural abscess (SEA) (Moustafa et al., 2019; Shin et al., 2022) and discitis (Pilgaard, 1969). However, few clinical cases of E.coli infection of lumbar bone tissue have been reported, and the mechanism is still unclear, as is the systematic comparison between M.tb and E.coli in cases of lumbar infection. To distinguish M.tb-induced lumbar spine infection from infection by other pathogenic bacteria, we used proteomics technology to compare the samples of lumbar spine samples infected with E.coli with STB samples for the first time. 

Our results showed that the reduction of mast cell particles, the inhibition of chondrocyte growth and development and the synthesis of the extracellular matrix led to the increase of inflammation and bone tissue damage. During the E.coli infection, the MFAP4 and CMA1 proteins with a down-regulation ratio were closely related to the synthesis of the extracellular matrix. As the only gene found in humans that encodes chymotrypsin, CMA1 directly affects ECM components of the extracellular matrix or indirectly affects processes related to ECM remodeling by activating ECM remodeling enzymes (Wernersson and Pejler, 2014; Kulkarni et al., 2022). Microfibrillar-associated protein 4 (MFAP4), an ECM protein belonging to the fibrinogen-associated domain family has functions that include stimulating cell proliferation, migration, inflammation and ECM deposition (Kanaan et al., 2022). In addition, MFAP4 promotes the activation of the TGF-β pathway, which plays a key role in pathological tissue remodeling (Kanaan et al., 2022). At the same time, GO enrichment analysis showed that the down-regulated differentially expressed proteins in SEcoli were significantly enriched in multiple ECM-related pathways, suggesting that one important way E.coli destroys bone tissue is by destroying the ECM structure and causing tissue damage.

Interestingly, the specificity of GSDME was found to be increased in SEcoli samples and was associated with pyroptosis and apoptosis. However, during E.coli infection, the process of cell apoptosis was inhibited, while pyroptosis-related pathways and proteins were not detected. Oxidative stress, the endogenous or exogenous apoptotic pathway, endoplasmic reticulum stress, the MAPK pathway, and other diverse conditions can lead to the activation of caspase-3 in cells expressing GSDME, leading to GSDME-dependent pyroptosis (Zhang et al., 2020; Shangguan et al., 2021; Liao et al., 2022). Through immunofluorescence, we further found that the expression of caspase-3 in SEcoli samples was significantly down-regulated, but there was no difference in STB samples, suggesting that E.coli inhibits host pyroptosis by inhibiting the expression of caspase-3, which could become a new therapeutic target for SEcoli.

Through a comparative analysis of two kinds of infection samples, the significantly differentially expressed proteins in STB mainly regulated muscle growth and development, while the major differentially expressed proteins in SEcoli mainly affected the biological processes related to ECM. Moreover, the bone tissue growth and development were found to be regulated by different proteins and factors. We compared and analyzed 88 identical differentially expressed proteins in the STB and SEcoli samples. We found that these differentially expressed proteins were all enriched in biological processes, with the down-regulated proteins mainly concentrated in the PI3K-Akt anti-apoptosis pathway. This suggests that in addition to M.tb, the pathway of activating apoptosis may provide new solutions for a variety of lumbar infections.

This study was the first to systematically compare spinal tuberculosis (STB) and E.coli lumbar spine infection (SEcoli) using 4D-label-free proteomics technology combined with immunohistochemistry and qRT-PCR validation. The integration of multiple techniques allowed us to comprehensively explore the molecular mechanisms of bone damage in STB and SEcoli infections and identify potential therapeutic targets such as THEMIS2. Another strength was the inclusion of both proteomics discovery and subsequent validation using fresh clinical samples, which improved the reliability of the findings. However, there were also several limitations. Due to the rarity of E. coli lumbar spine infections, the SEcoli sample size was relatively small, which might limit the generalizability of the findings. In addition, the use of paraffin-embedded tissues for proteomic analysis, although practical in clinical settings, might introduce bias in protein extraction and quantification. Unfortunately, due to the difficulty of obtaining sufficient samples, our study did not analyze the potential impact of different age groups on the pathogenic mechanisms of spinal tuberculosis and E. coli bone infections. This remains an important direction for future research. In future studies, larger sample sizes and analyses are warranted to further validate and expand on these results.

## Data Availability

All data generated or analyzed during this study are included in this article or uploaded as **Supplementary Information**.

## Supplementary material

Supplementary Table 1 Sample information.

Supplementary Table 2 Primer sequences.

Supplementary Table 3 qRT-PCR program.

Supplementary Figure 1 Lumbar STB. **(A)** MRI image showed damage to the L2 and L3 vertebrae and the L2/3 disc, along with neurological compression within the red box; **(B)** A Ziehl-Neelsen stain of a bone tissue section with acid-fast bacilli marked within the red box.

Supplementary Figure 2 Immunohistochemical analysis of CD68 in granuloma tissue. **(A–C)** come from three different patients. The magnification is 200 ×, the blue-stained regions are the nuclei, and the brown-yellow stained regions are the CD68-positive area.
