# Supplementary material for: Molecular mechanisms related to bone damage in spinal tuberculosis revealed by 4D-label-free proteomics analysis
Source: Front Cell Infect Microbiol. 2025 Sep 22;15:1629805. doi: 10.3389/fcimb.2025.1629805 (PMC12497785; doi:10.3389/fcimb.2025.1629805)
Supplement: Supplementary Table 1 — Sample information. [file DataSheet1.docx]

**Supplement**

**Table S1. Samples information**

| 4D-FQ group for **STB** | 4D-FQ group for **SEcoli** | ID | Sex | Age (years) | Disease description | Clinical symptoms | Pregnant | HIV |
| --- | --- | --- | --- | --- | --- | --- | --- | --- |
| STB infection group |  | A | Male | 64 | Infection at L1/2 | Low back pain | No | Negative |
|  |  | B | Female | 24 | Infection at T12, L1 | Low back pain | No | Negative |
|  |  | C | Female | 76 | Infection at L1/2 | Low back pain | No | Negative |
| LDD control group | LDD control group | D | Female | 39 | L5 vertebral spondylolisthesis | Low back pain | No | Negative |
|  |  | E | Female | 60 | Lumbar disc herniation | Low back and leg pain | No | Negative |
|  |  | F | Male | 64 | L1 vertebral fracture | Low back pain | No | Negative |
|  |  | G | Female | 57 | Lumbar disc herniation, L4 vertebral spondylolisthesis | Low back and leg pain | No | Negative |
|  |  | H | Male | 60 | Lumbar disc herniation | Low back pain | No | Negative |
|  | SEcoli infection group | I | Female | 50 | Infection at L3/4 | Low back pain | No | Negative |
|  |  | J | Female | 75 | Infection at L3/4/5 | Low back pain | No | Negative |


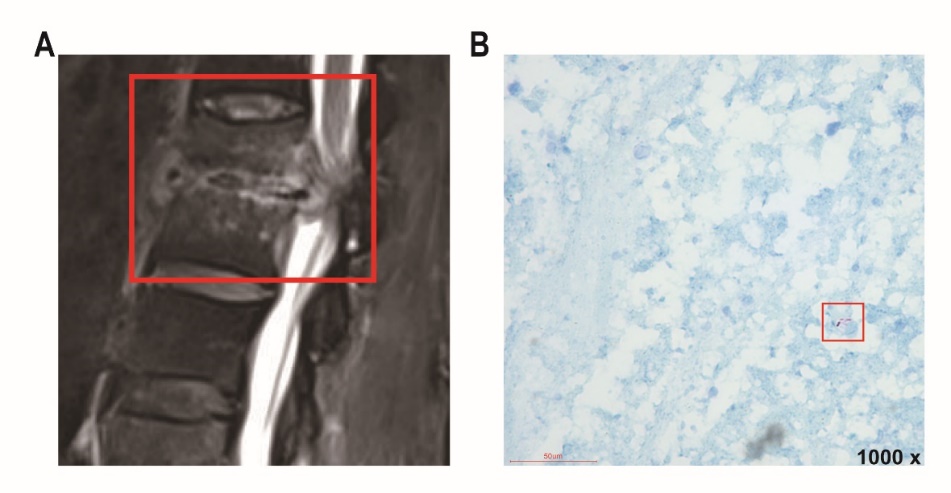


**Figure S1. Lumbar STB.** (A) MRI image showed damage of L2, L3 vertebra and L2/3 disc, and neurologic compression inside the red box; (B) Ziehl-Neelsen of bone tissue section, acid-fast bacilli are marked in the red box.


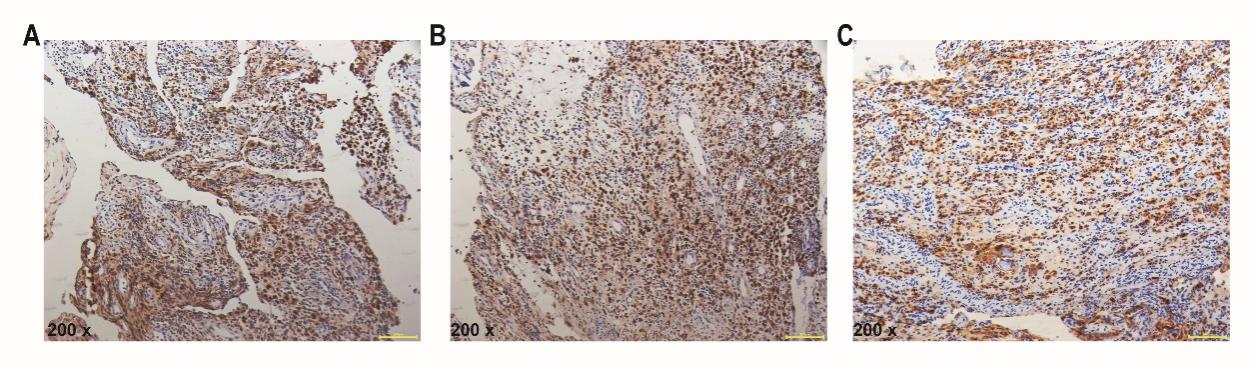


**Figure S2. Immunohistochemistry analysis of CD68 in granulation tissues**. (A), (B), (C) comes from three different patients. Magnification is 200 ×, blue staining regions are nuclei, brown-yellow staining regions are CD68 positive area.
